# Methanol Dehydrogenases as a Key Biocatalysts for Synthetic Methylotrophy

**DOI:** 10.3389/fbioe.2021.787791

**Published:** 2021-12-24

**Authors:** Thien-Kim Le, Yu-Jin Lee, Gui Hwan Han, Soo-Jin Yeom

**Affiliations:** ^1^ School of Biological Sciences and Technology, Chonnam National University, Gwangju, South Korea; ^2^ School of Biological Sciences and Biotechnology, Graduate School, Chonnam National University, Gwangju, South Korea; ^3^ Center for Industrialization of Agricultural and Livestock Microorganisms (CIALM), Jeollabuk-do, South Korea

**Keywords:** methanol dehydrogenase, synthetic methylotrophy, C1 gas, assimilation, formaldehyde

## Abstract

One-carbon (C1) chemicals are potential building blocks for cheap and sustainable re-sources such as methane, methanol, formaldehyde, formate, carbon monoxide, and more. These resources have the potential to be made into raw materials for various products used in our daily life or precursors for pharmaceuticals through biological and chemical processes. Among the soluble C1 substrates, methanol is regarded as a biorenewable platform feedstock because nearly all bioresources can be converted into methanol through syngas. Synthetic methylotrophy can be exploited to produce fuels and chemicals using methanol as a feedstock that integrates natural or artificial methanol assimilation pathways in platform microorganisms. In the methanol utilization in methylotrophy, methanol dehydrogenase (Mdh) is a primary enzyme that converts methanol to formaldehyde. The discovery of new Mdhs and engineering of present Mdhs have been attempted to develop synthetic methylotrophic bacteria. In this review, we describe Mdhs, including in terms of their enzyme properties and engineering for desired activity. In addition, we specifically focus on the application of various Mdhs for synthetic methylotrophy.

## Introduction

One-carbon (C1) substrates are potential feedstocks and have recently gained attention and preference in industrial fields due to their natural abundance, low production cost, and availability as industrial by-products (Jiang et al., 2021). Among C1 chemicals, methanol is a potentially renewable feed stock for microorganisms as it is electron rich and can be derived from methane or CO_2_ (Chen et al., 2020). In nature, methylotrophs, such as *Methylobacterium extorquens* and *Bacillus methanolicus*, can utilize methanol, and their biochemical function have been characterized (Brautaset et al., 2007; Bennett et al., 2018). However, so far, there are limitations in the engineering of native methylotrophs to produce heterologous products at high rates and titers due to the lack of genetic tools available. Recent advances in synthetic biology, integration of efficient methanol converting enzymes, genome engineering, and laboratory evolution are enabling the first steps toward the creation of synthetic methanol-utilizing microorganisms (Heux et al., 2018; Meyer et al., 2018; Bennett et al., 2020; Chen et al., 2020; Keller et al., 2020; Wang et al., 2020).

**FIGURE 1:**
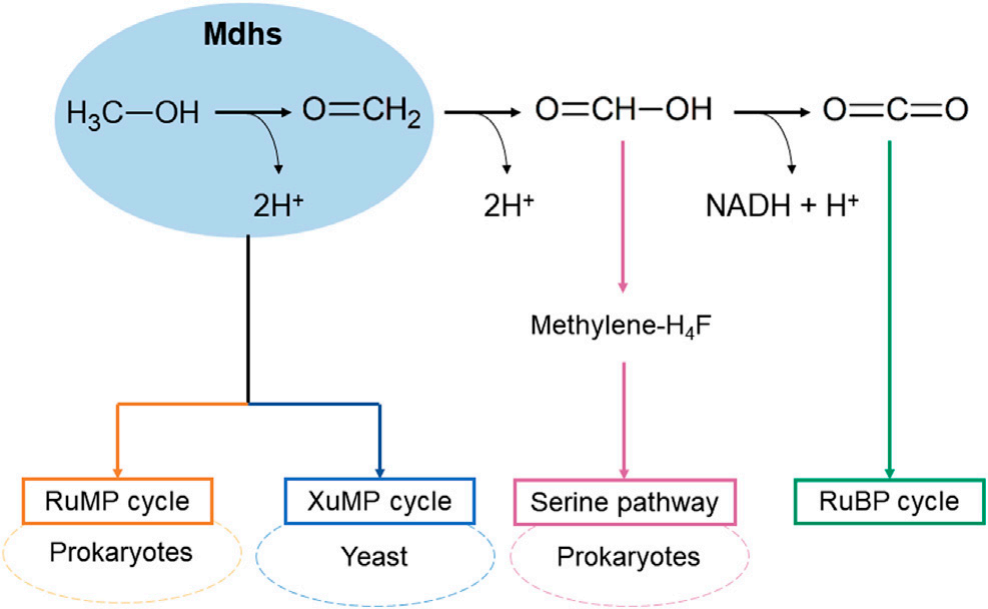
Natural methane and methanol utilization pathways in methylotrophs.

In the methanol utilization in methylotrophy, one of the key steps is the oxidation of methanol to formaldehyde by oxidoreductase (Zhang et al., 2017), and methanol dehydrogenases (Mdhs) are the main enzymes as they catalyze the oxidation of methanol to form formaldehyde with two electrons and 2H^+^ (Le et al., 2021) (Figure 1). There are three native pathways of formaldehyde assimilation, that have been discovered and biochemically described for growth support of microorganisms in methanol, as follows: the ribulose monophosphate (RuMP) cycle, serine pathway, and xylulose monophosphate (XuMP) cycle (Figure 1) (Zhang et al., 2017). The RuMP and serine cycles mainly occur in prokaryotes, the XuMP cycle is found in yeasts. Among them, the RuMP cycle of hexulose-6-phosphate synthases (HPS) and 6-phospho-3-hexulose isomerase (PHI) has been identified as the best combination because of its highest theoretical growth rate; thus, it has received the most attention (Heux et al., 2018; Claassens et al., 2019). Meanwhile, there have been a modified serine cycle in *Escherichia coli* was reported (Yu and Liao 2018) and only one study on XuMP in *Saccharomyces cerevisiae* (Dai et al., 2017).

Various hypotheses have been proposed regarding potential bottlenecks to efficient methanol assimilation. In particular, the concentration of Mdhs is a limitation, and poor kinetic and thermodynamic properties of methanol oxidation by nicotinamide adenine dinucleotide (NAD)- Mdh is widely acknowledged (Whitaker et al., 2017; Woolston et al., 2018). The low activity and substrate affinity of Mdh fundamentally limits methanol assimilation flux, while a high NADH/NAD^+^ ratio negatively impacts the Gibbs free energy of methanol oxidation (Wang et al., 2020). Thus, the development of efficient Mdhs presents a significant challenge to synthetic methylotrophy. In this review, we summarize the current classifications, enzyme properties, and engineering of reported Mdhs. Additionally, we provide a comprehensive overview of recent advances in the use of Mdhs in engineering synthetic methylotrophy.

### Class of Methanol Dehydrogenases

**FIGURE 2:**
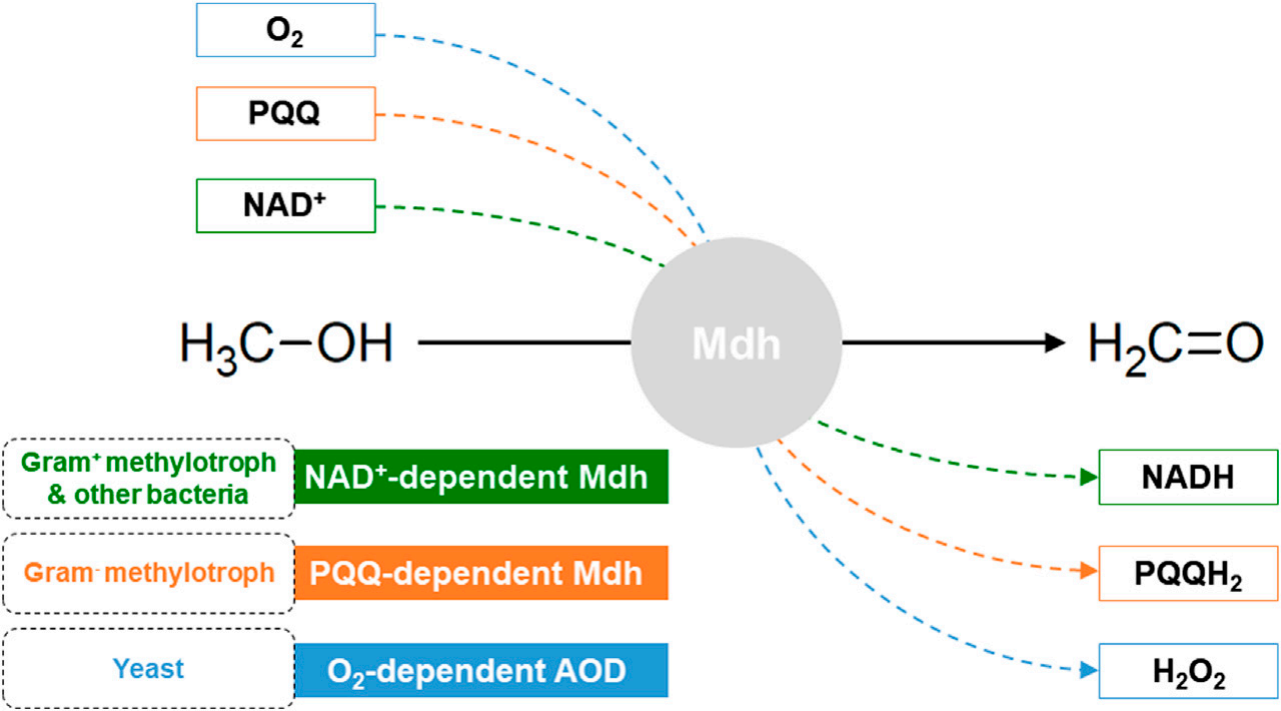
Three classes of methanol dehydrogenases (Mdhs).

Depending on the electron acceptors, Mdhs in methylotrophs are classified into three groups: NAD^+^-dependent Mdh, PQQ (pyrrolo-quinoline quinone)-dependent Mdh, and O_2_-dependent **AOX** (alcohol oxidase) (Figure 2).

### NAD^+^-dependent Mdh

NAD^+^-dependent Mdh in thermophilic Gram-positive methylotrophs uses NAD^+^ as the cofactor for the methanol oxidation. The first NAD^+^-dependent Mdh was reported in 1989 (Arfman et al., 1989). NAD^+^-dependent Mdhs also obtained from non-methylotrophic bacteria. To date, several NAD^+^-dependent Mdhs have been isolated from *Bacillus* sp. (such as *B. methanolicus* (Arfman et al., 1989; Arfman et al., 1991; Müller et al., 2015; Witthoff et al., 2015; Price et al., 2016) and *B. stearothermophilus* (Whitaker et al., 2017)), *Lysinibacillus* sp. (such as *L. xylanilyticus* (Lee et al., 2020)), and *Cupriavidus* sp. (such as *C. necator* (Wu et al., 2016)). In particular, their NAD^+^-dependent Mdhs have been focused and reported for studies of recombinant *E. coli* as synthetic methylotrophs (Müller et al., 2015; Wu et al., 2016; Whitaker et al., 2017; Lee et al., 2020; Le et al., 2021). Three NAD^+^-dependent Mdhs have been found in *B. methanolicus* MGA3 (Mdh, Mdh2, and Mdh3). Moreover, the activity of all three Mdhs is modulated by an endogenous Mdh activator protein (ACT). *In vitro* studies suggest that ACT enhances the methanol affinity, oxidation rate, and catalytic activity of Mdhs; however, the detailed mechanism for activation is currently unclear (Hektor et al., 2002; Witthoff et al., 2015) and no effect has been shown *in vivo* in a synthetic methylotrophy (Müller et al., 2015) because detail research for the activator protein functions in native host has not been tested. To enable the assimilation of methanol as the carbon source in metabolic engineering, ACT-independent Mdhs and their mutants from *C. necator* (Wu et al., 2016; Chen et al., 2018) and *L. xylanilyticus* (Lee et al., 2020; Le et al., 2021) have been reported and introduced into *E. coli* for methanol assimilation. As best candidate for synthetic methylotrophy, NAD^+^-dependent Mdh that can perform its function under both aerobic and anaerobic conditions (Zhang et al., 2017). Besides, it uses NAD^+^, which is ubiquitous and can provide electrons for metabolite products, as the cofactor. Therefore, it may be the best candidate for recombinant-based synthetic methylotrophs (Zhang et al., 2017).

### PQQ-dependent Mdh

In Gram-negative methylotrophs, the oxidation of methanol occurs in the periplasmic space by PQQ-dependent Mdh (Skovran et al., 2019). Pure PQQ-dependent Mdh was first described in 1967 (Anthony and Zatman 1967). To date, PQQ-dependent Mdh has been isolated and purified from several different strains of microorganisms including *Pseudomonas* sp. (Anthony and Zatman 1965; Anthony and Zatman 1967; Patel et al., 1972), *Methylococcus capsulatus* (Patel et al., 1972), *Hyphomicrobium denitrificans* (Nojiri et al., 2006), *Methylorubrum extorquens* (formerly *Methylobacterium extorquens*) (Anthony 2004; Liu et al., 2006; Nakagawa et al., 2012), *Methyloversatilis universalis* FAM5 (Kalyuzhnaya et al., 2008), *Methylibium petroleiphilum* (Kalyuzhnaya et al., 2008), *Methylophaga aminisulfidivorans* (Kim et al., 2012; Cao et al., 2018), *Methylobacterium nodulans* (Kuznetsova et al., 2012), *Methylophilus* sp. (Leopoldini et al., 2007; Li et al., 2011), *Burkholderiales* sp. (Kalyuzhnaya et al., 2008), *Paracoccus denitrificans* (Xia et al., 2003), *M. radiotolerans* (Hibi et al., 2011), *Bradyrhizobium* sp. (Fitriyanto et al., 2011), *M. aquaticum* (Masuda et al., 2018), *Methylomicrobium buryatense* (Deng et al., 2018), *M. fumariolicum* (Jahn et al., 2018), and *Bradyrhizobium diazoefficiens* (Wang et al., 2019). The PQQ-dependent Mdh contains a PQQ prosthetic group. The chemical structure of the PQQ prosthetic group has been confirmed by two independent research groups using a wide range of chemical and physical techniques, such as X-ray, UV/Vis absorption spectra, and HPLC (Anthony 1982). The role of the PQQ prosthetic group is capturing electrons from methanol oxidation and passing them to the cytochrome (Anthony 2004). The biggest disadvantage is the requirement of molecular oxygen for PQQ bio-synthesis (Velterop et al., 1995), while some desired intermediates as precursors of value-added products such as lactate must be produced under anaerobic conditions. Therefore, this limits the application of PQQ-dependent Mdhs.

In genomes of methylotrophs, PQQ-dependent Mdhs are generally encoded by MxaFI and XoxF. MxaFI consists of small (MxaI) and large (MxaF) subunits, encoding PQQ-dependent Mdh using calcium (Ca^2+^) as a cofactor (MxaFI-Mdh) (Anthony 2004). Another PQQ-dependent Mdh, which uses lanthanides (Ln^3+^) instead of Ca^2+^, is encoded by XoxF (XoxF-type Mdh) (Chistoserdova 2016; Skovran et al., 2019). XoxF-Mdh from *M. extorquens* AM1 is a representative of Ln^3+^-dependent Mdh that it was studied carefully to show the biochemical characterization. XoxF of M. extorquens AM1 showed better activity when La^3+^ or Ca^2+^ and La^3+^ were added together than when Ca^2+^ was used alone as part of the cofactor complex (Vu et al., 2016; Good et al., 2020). In addition, other elements of lanthanide (Ce^3+^, Nd^3+^, Pr^3+^, Sm^3+^, Eu^3+^, or Gd^3+^) were also found to be involved in the methanol oxidation activity (Pol et al., 2014). Lanthanides as important factor was suggested in regulatory and catalytic functions because the XoxF genes are required for transcription of the MxaFI (Vu et al., 2016).

### O_2_-dependent AOX

Unlike NAD^+^-dependent and PQQ-dependent Mdhs, O_2_-dependent **AOX** is obtained from eukaryotic methylotrophs and is located in the peroxisome of yeasts (Egli et al., 1980). First, formaldehyde and hydrogen peroxide (H_2_O_2_), which are highly toxic chemicals for cells, are created from methanol oxidation by O_2_-dependent **AOX**. To protect the cells, dihydroxyacetone synthase (DAS) and catalase (CTA) work to transform them into non-toxic chemicals (Zhang et al., 2017). O_2_-dependent **AOX** only function under aerobic conditions and thus, has limitations similar to those of PQQ-dependent Mdh. In addition, another important limitation AOX’s is that the electrons from methanol are not captured as useable energy by the cell, but wasted in the generation of peroxide.

## Biochemical Characterization of Methanol Dehydrogenases

Among three classes of Mdhs, enzyme properties of NAD^+^- and PQQ-dependent Mdhs are summarized in Table 1.

**TABLE 1:** Enzyme properties of NAD^+^-Dependent Mdhs (EC number: 1.1.1.244) and PQQ-Dependent Mdhs (EC number: 1.1.2.7).

Enzyme	Source	Optimum tem. (°C)	Optimum pH	Molecular weight (kDa)		Association form	Metal ion	Refs
				Subunit	Native			
NAD^+^-Dependent Mdhs	*Bacillus methanolicus* C1	57–59	9.5	4.3	43	Decamer	Mg^2+^	Arfman et al. (1991)
	*Bacillus methanolicus* MGA3	37	7.4	N.I.	43	Decamer	Mg^2+^	Müller et al. (2015)
		45	9.5	N.I.	N.I.	Decamer	Mg^2+^	Krog et al. (2013)
		50	9.5	N.I.	43	Decamer	Mg^2+^	Witthoff et al. (2015)
		50	9.0	N.I.	N.I.	Decamer	Mg^2+^	Ochsner et al. (2014)
	*Bacillus methanolicus* PB1	37	7.4	N.I.	43	Decamer	Mg^2+^	Müller et al. (2015)
		45	9.5	N.I.	N.I.	Decamer	Mg^2+^	Krog et al. (2013)
	*Bacillus stearothermophilus*	37	7.4	N.I.	N.I.	N.I.	Mg^2+^	Whitaker et al. (2017)
	*Lysinibacillus xylanilyticus*	55	9.5	N.I.	42.8	N.I.	Mg^2+^	Lee et al. (2020)
	*Cupriavidus necator* N-1	30	9.5	N.I.	40.7	N.I.	Ni^2+^	Wu et al. (2016)
PQQ-Dependent Mdhs	*Pseudomonas* sp. M27	N.I.	9.0	α: 62, β: N.I.	120	N.I.	N.I.	Patel et al. (1972)
	*Methylococcus capsulatus* (Texas strain)	N.I.	9.0	α: 62, β: N.I.	120	N.I.	N.I.	
	*Hyphomicrobium denitrificans* A3151	25	7.0	α: 65, β: 9	148	Heterotetramer	N.I.	Nojiri et al. (2006)
	*Methylorubrum extorquens*	N.I.	7.0	α: 66, β: 8.5	149	Heterotetramer	Ca^2+^	Anthony (2004)
	*Methylorubrum extorquens* AM1	N.I.	9.0	α: 62, β: 7.5	139	Heterotetramer	Ca^2+^	Liu et al. (2006)
		30	8.0	N.I.	117	Homodimer	La^3+^	Nakagawa et al. (2012)
		N.I.	8.0	N.I.	N.I.	N.I.	La^3+^, Nd^3+^	Good et al. (2019)
		N.I.	8.0	N.I.	N.I.	N.I.	Gd^3+^	Good et al. (2021)
	*Methyloversatilis universalis* FAM5	22	7.5	α: 65, β: N.I.	N.I.	Monomer	N.I.	Kalyuzhnaya et al. (2008)
	*Methylibium petroleiphilum* PM1	22	7.5	α: 65, β: N.I.	N.I.	Monomer	N.I.	Kalyuzhnaya et al. (2008)
	*Burkholderiales* strains Z18-153	R.T	8.8	α: 65, β: N.I.	N.I.	Monomer	N.I.	Kim et al. (2012)
	*Burkholderiales* strains FAM1	R.T	8.8	α: 65, β: N.I.	N.I.	Monomer	N.I.	
	*Methylophaga aminisulfidivorans* MP^T^	30	8.0	α: 65.98, β: 7.58	147.12	Tetramer	Ca^2+^	
	*Methylophaga aminisulfidivorans* MP^T^ Mdh_*Mas*_	N.I.	N.I.	α: 65, β: 7.5	145	Heterotetramer	Mg^2+^	Cao et al. (2018)
	*Methylobacterium nodulans* ORS 2060T	50	9–10	α: 60, β: 6.5	70	Heterodimer	No metal	Kuznetsova et al. (2012)
	*Methylophilus methylotrophus* W3A1	N.I.	N.I.	α: 62, β: 8	140	Heterotetramer	Ca^2+^	Leopoldini et al. (2007)
	*Paracoccus denitrificans*	N.I.	N.I.	α: 67, β: 9.5	153	Heterotetramer	Ca^2+^	Xia et al. (2003)
	*Methylobacterium radiotolerans* NBRC15690	N.I.	N.I.	α: 63, β: N.I.	120	Homodimer	La^3+^	Hibi et al. (2011)
	*Methylobacterium radiotolerans* NBRC15690	N.I.	N.I.	α: 60, β: 10	114	Heterotetramer	Ca^2+^	Hibi et al. (2011)
	*Bradyrhizobium* sp. MAFF211645	N.I.	N.I.	α: 68, β: N.I.	108	Homodimer	Ce^3+^	Fitriyanto et al. (2011)
	*Methylobacterium aquaticum* strain 22A	N.I.	N.I.	N.I.	N.I.	N.I.	La^3+^	Masuda et al. (2018)
	*Methylomicrobium buryatense* 5GB1C	N.I.	N.I.	α: 67.2, β: N.I.	N.I.	Homodimer	La^3+^	Deng et al. (2018)
	*Methylacidiphilum fumariolicum* SolV	45	7.2	N.I.	63.6	Homodimer	Eu^3+^	Jahn et al. (2018)
	*Bradyrhizobium diazoefficiens* strain USDA110	N.I.	N.I.	α: 64, β: N.I.	136	N.I.	Ce^3+^	Wang et al. (2019)

R.T—Room temperature; N.I—No information.

### Optimal Conditions for Methanol Oxidation Reaction by Mdhs

The most important factor, which has a considerable effect on the activity of Mdhs, is cofactor binding. For NAD^+^-dependent Mdhs, a metal ion is involved in cofactor binding which may influence enzymatic activity (Hektor et al., 2002). Several metal ions have been examined for the effects on the methanol oxidation activity of Mdhs, such as Fe^2+^, Mn^2+^, Zn^2+^, Cu^2+^, Co^2+^, Ni^2+^, or Mg^2+^ ions (Sridhara et al., 1969; Arfman et al., 1991; Montella et al., 2005; Müller et al., 2015; Wu et al., 2016; Whitaker et al., 2017; Lee et al., 2020; Le et al., 2021). In general, the supplementation of Fe^2+^ or Mn^2+^ ions increase enzyme activity, and Mdh activity is inhibited by Cu^2+^, Co^2+^, or Zn^2+^ (Sridhara et al., 1969; Montella et al., 2005). In the case of Mdhs from *Lysinibacillus xylanilyticus* (Lxmdh), Mn^2+^ or Fe^2+^ reduce its activity, whereas and Zn^2+^, Cu^2+^, or Co^2+^ inhibit Lxmdh activity (Lee et al., 2020). For almost all NAD^+^-dependent Mdhs, Mg^2+^ increases the effect of enzyme activity (Arfman et al., 1989; Arfman et al., 1991; Müller et al., 2015; Whitaker et al., 2017; Lee et al., 2020; Le et al., 2021). For Mdhs from *Cupriavidus necator* (Cnmdh), Ni^2+^ is typically the chosen cofactor (Wu et al., 2016).

For PQQ-dependent Mdhs, Ca^2+^ plays a role in the active site (Anthony and Zatman 1967; Anthony 2004). The X-ray structure of Mdhs from *M. extorquens*, *M. nodulans*, *Methylophilus* sp, and *P. denitrificans* has been determined to have one molecule of PQQ and one Ca^2+^ ion in each large α-subunit, which is encoded by MxaF (Anthony and Williams 2003; Anthony 2004). Moreover, some types of Mdhs, which are encoded by XoxF, use Ln^3+^ instead of Ca^2+^, which is a part of cofactor complex for Mdhs encoded by MxaF (Egli et al., 1980; Skovran et al., 2019). Ln^3+^ was first suggested as a metal ion of the cofactor complex for PQQ-dependent Mdhs obtained from *M. radiotolerans* (Hibi et al., 2011) and *Bradyrhizobium* sp. (Fitriyanto et al., 2011) in 2011. Furthermore, Mdhs from *M. extorquens* AM1 (Nakagawa et al., 2012), *M. aquaticum* (Masuda et al., 2018), *M. buryatense* (Deng et al., 2018), *M. fumariolicum* (Jahn et al., 2018), and *B. diazoefficiens* (Wang et al., 2019) have been observed to be Ln^3+^-dependent Mdhs. Interestingly, the subunits of PQQ-dependent Mdh from *M. aminisulfidivorans* MP^T^ are coordinated by an Mg^2+^ ion instead of a Ca^2+^ ion or Ln^3+^ group (Cao et al., 2018). In addition, the activity of PQQ-dependent Mdhs under aerobic conditions with artificial electron acceptors *in vitro* requires the presence of an activator (e.g., ammonium salt) (Anthony 1982; Anthony and Williams 2003; Anthony 2004; Kuznetsova et al., 2012).

The other most important factors are the temperature and pH of the buffer in the enzyme assay. Almost all methanol dehydrogenases have high activity at high temperatures (∼55°C) and high pH (9–10). Mdhs from *M. nodulans* (Mnmdh) exhibits maximal activity at pH 9–10, and it increases linearly with increasing temperature from 20°C to 50°C (Kuznetsova et al., 2012). The optimum pH for Mdh from *Pseudomonas* sp. M27 (Patel et al., 1972), *M. capsulatus* (Patel et al., 1972), and *M. extorquens* AM1 (Liu et al., 2006) are also 9. Similarly, an assay involving NAD^+^-dependent Mdhs from thermotolerant methylotrophic *Bacillus* strains is performed at 45–50°C, using glycine/KOH buffer at pH 9.5 (Arfman et al., 1991; Krog et al., 2013). Lxmdh and its mutant or Cnmdh also function better in buffers with a pH of 9.5; however, Lxmdh and its mutant exhibit high activity at 55°C (Lee et al., 2020; Le et al., 2021), while the temperature for testing Cnmdh activity is 30°C (Wu et al., 2016). On the other hand, the conditions for the Mdhs from *M. methanolicus* (Bmmdh) and *B. stearothermophilus* (Bsmdh) reactions are similar, at pH 7.4 and 37°C (Müller et al., 2015; Whitaker et al., 2017). When examining the activity of Mdhs obtained from *L. xylanilyticus* or *Burkholderiales* using spectrometer experiments to detect the changes in absorbance, room temperature is preferred (Kalyuzhnaya et al., 2008; Le et al., 2021). Moreover, buffer systems with a pH of 8.8 are used for *Burkholderiales* Mdh assays (Kalyuzhnaya et al., 2008). On the whole, the Mdh assay requires the presence of an ion as the binding cofactor. This depends on the type and source of Mdh. For PQQ-dependent Mdhs, the activator for enzyme activity is required under aerobic conditions.

### Molecular Weight of Methanol Dehydrogenases

The molecular weight of most PQQ-dependent Mdhs has been identified as being between 112 and 158 kDa. The associated form of almost all PQQ-dependent Mdhs, which are Ca^2+^-dependent Mdhs, is a tetramer (α_2_β_2_). Therefore, it can be dissociated to α-subunits (56–76 kDa) and β-subunits (very small, ≤ 10 kDa) by a low pH or sodium dodecyl sulfate (SDS) (Anthony 1982), such as the Mdh from *H. denitrificans* (α: 65 kDa, β: 9 kDa) (Nojiri et al., 2006), *M. extorquens* (α: 62–65 kDa, β: 7.5–8.5 kDa) (Anthony 2004; Liu et al., 2006), *M. aminisulfidivorans* MP^T^ (α: 65–66 kDa, β: 7.5–7.6 kDa) (Kim et al., 2012; Cao et al., 2018), *M. methylotrophus* (α: 62 kDa, β: 8 kDa) (Leopoldini et al., 2007; Li et al., 2011), and *M. radiotolerans* (α: 60 kDa, β: 10 kDa) (Hibi et al., 2011). There are also some special cases with the heterodimer form (αβ), for example, Mdh from *M. nodulans* (α: 60 kDa, β: 6.5 kDa) (Kuznetsova et al., 2012). Besides, the associated form of La^3+^-dependent Mdhs is a homodimer (formed by two identical proteins), e. g, Mdhs from *M. radiotolerans* (120 kDa) (Hibi et al., 2011), *Bradyrhizobium* sp. (108 kDa) (Fitriyanto et al., 2011), *M. extorquens* AM1 (117 kDa) (Nakagawa et al., 2012), *Methylacidiphilum fumariolicum* SolV (63.6 kDa) (Jahn et al., 2018), and *M. buryatense* (Deng et al., 2018). On the other hand, the NAD^+^-dependent Mdh with a single subunit has a molecular weight of around 40 kDa. For instance, the molecular weight of NAD^+^-dependent Mdh from *Bacillus* sp. C1 (a thermotolerant methylotrophic *Bacillus*) is 43 kDa (Arfman et al., 1989; Arfman et al., 1991). Other *B. methanolicus* strains (MGA3 and PB1) show a similar molecular weight at 43 kDa (Müller et al., 2015; Witthoff et al., 2015; Price et al., 2016). Moreover, Cnmdh from *C. necator* N-1 (Wu et al., 2016) or Lxmdh from *L. xylanilyticus* (Lee et al., 2020) show respective molecular subunits at 40.7 or 42.8 kDa (Table 1). According to the previous report, NAD^+^-dependent Mdhs has decameric association structure (430 kDa) as native form (Vonck et al., 1991).

### Substrate Affinity Toward Methanol of Wild-type or Engineered NAD-Mdh

Although, MxaFI-Mdhs from *M. extorquens* AM1, with a high efficiency (*k*_cat_/*K*_M_) of methanol production, has been suggested as the best choice for engineering *E. coli* (Anthony and Williams 2003), it requires at least 11 gene products for its functional assembly (Chistoserdova et al., 2003). In addition, XoxF-Mdhs from *M. extorquens* AM1 would be required only three genes with a high catalytic efficiency (Keltjens et al., 2014), PQQ-dependent Mdhs are not suitable for synthetic methylotrophy using engineered *E. coli*. Because, PQQ as critical cofactor is critical limit that specially *E. coli* is not able to synthesize PQQ (Anthony 2004). In the case of O_2_-dependent **AOX**, its product, H_2_O_2_, is also challenging because it is the highly toxic to most hosts. Therefore, only NAD^+^-dependent Mdh has been considered as the best candidate for synthetic methylotrophs (Zhang et al., 2017), which requires only one gene for functional production and can generate the reducing equivalent (NADH) to promote strain growth under both aerobic and anaerobic conditions. To successfully achieve methanol assimilation, the Mdh kinetics, including substrate affinity and catalytic activity, should be improved for methanol assimilation through directed evolution or rational approach based engineering. Various NAD^+^-dependent Mdhs from *B. methanolicus* (Vonck et al., 1991; De Vries et al., 1992; Hektor et al., 2002; Krog et al., 2013; Ochsner et al., 2014; Müller et al., 2015; Witthoff et al., 2015), *C. necator* (Wu et al., 2016), *B. stearothermophilus* (Whitaker et al., 2017), *L. xylanilyticus* (Lee et al., 2020) were reported in methanol conversion. Researchers are searching for NAD^+^-dependent Mdhs with higher activity and lower *K*_M_ from different microorganisms and improving their characteristics by a rational approach and directed evolution (Hektor et al., 2002; Ochsner et al., 2014; Roth et al., 2019; Lee et al., 2020; Le et al., 2021) (Table 2). Specially, the improvement of substrate affinity toward low concentration methanol is focused in the development of Mdh-driven synthetic methylotrophy because of the high toxicity of methanol for *E. coli* (Dyrda et al., 2019).

**TABLE 2:** Summary of substrate affinity for methanol by NAD^+^-Dependent Mdhs.

Enzyme type	Strain	Type of enzyme	*V*_max_ (U/mg)	*k*_cat_ (s^−1^)	*K*_M_ (mM)	Evolution method	Refs
*Wild type Mdh*	*B. methanolicus* MGA3	*Mdh*	0.06 ± 0.002	N.I.	170 ± 20	WT	Krog et al. (2013)
		*Mdh* 2	0.09 ± 0.003	N.I.	360 ± 30	WT	
		*Mdh* 3	0.07 ± 0.005	N.I.	200 ± 70	WT	
		*Mdh* + ACT	0.4 ± 0.02	N.I.	26 ± 7	WT	
		*Mdh* 2 + ACT	0.2 ± 0.008	N.I.	200 ± 20	WT	
		*Mdh* 3 + ACT	0.4 ± 0.008	N.I.	150 ± 10	WT	
		*Mdh*	0.151 ± 0.008	0.11 ± N.I.	150 ± 25	WT	Ochsner et al. (2014)
		*Mdh* 2	0,151 ± 0.012	0.12 ± N.I.	416 ± 97	WT	
		*Mdh* + ACT	0.474 ± 0.032	0.32 ± N.I.	9 ± 2	WT	
		*Mdh* 2 + ACT	0.394 ± 0.016	0.27 ± N.I.	96 ± 12	WT	
	*B. methanolicus* PB1	*Mdh*	0.03 ± 0.001	N.I.	220 ± 30	WT	Krog et al. (2013)
		*Mdh* 1	0.015 ± 0.001	N.I.	170 ± 60	WT	
		*Mdh* 2	0.08 ± 0.004	N.I.	330 ± 0.05	WT	
		*Mdh* + ACT	0.2 ± 0.003	N.I.	10 ± 1	WT	
		*Mdh* 1 + ACT	0.05 ± 0.002	N.I.	5 ± 1	WT	
		*Mdh* 2 + ACT	0.38 ± 0.04	N.I.	110 ± 50	WT	
	*C. necator* N-1 WT	*Mdh* 2	0.32 ± N.I.	0.22 ± 0.01	132 ± 15.4	WT	Wu et al. (2016)
	*B. stearothermophilus*	*Mdh*	2.1 ± N.I.	N.I.	20 ± N.I.	WT	Whitaker et al. (2017)
	*L. xylanilyticus*	*Mdh* 2	0.3027 ± 0.0169	0.21 ± 0.01	3.23 ± 1.05	WT	Lee et al. (2020)
*Engineered Mdh*	*B. methanolicus* MGA3 S98G	*Mdh*	0.44 ± 0.053	0.35 ± N.I.	1,151 ± 274	Rational approach	Ochsner et al. (2014)
	*B. methanolicus* MGA3 S98G + ACT	*Mdh*	0.819 ± 0.082	0.59 ± N.I.	847 ± 190	Rational approach	
	*C. necator* N-1 CT4-1	*Mdh* 2	0.29 ± N.I.	0.20 ± 0.01	21.6 ± 1.5	Directed evolution	Wu et al. (2016)
	*L. xylanilyticus Mdh* -S101V	*Mdh* 2	0.3423 ± 0.02167	0.24 ± 0.01	10.35 ± 3.87	Rational approach	Lee et al. (2020)
	*L. xylanilyticus Mdh* -T141S	*Mdh* 2	0.4629 ± 0.0576	0.33 ± 0.04	51.24 ± 23.95	Rational approach	
	*L. xylanilyticus Mdh* -A164F	*Mdh* 2	0.4753 ± 0.05072	0.33 ± 0.03	36.83 ± 15.82	Rational approach	
	*L. xylanilyticus Mdh* -E396V	*Mdh* 2	N.I.	0.020 ± 0.002	0.010 ± 0.003	Directed evolution	Le et al. (2021)
	*L. xylanilyticus Mdh* -K318N	*Mdh* 2	N.I.	0.027 ± 0.005	0.046 ± 0.072	Directed evolution	
	*L. xylanilyticus Mdh* -E396V + K318N	*Mdh* 2	N.I	0.022 ± 0.002	0.233 ± 0.107	Directed evolution	
	*B. methanolicus* (WT)	*Mdh* 2	0.0365 ± 0.0017	N.I.	636 ± 74	Directed evolution	Roth et al. (2019)
	*B. methanolicus* Q5L E123G	*Mdh* 2	0.0366 ± 0.0016	N.I.	615 ± 66	Directed evolution	
	*B. methanolicus* Q5L M163V	*Mdh* 2	0.055 ± 0.0031	N.I.	627 ± 89	Directed evolution	
	*B. methanolicus* Q5L A164P	*Mdh* 2	0.0754 ± 0.0023	N.I.	440 ± 39	Directed evolution	
	*B. methanolicus* Q5L A363L	*Mdh* 2	0.127 ± 0.0033	N.I.	432 ± 32	Directed evolution	
	*B. methanolicus* Q5L A164P A363L	*Mdh* 2	0.0885 ± 0.0023	N.I.	329 ± 28	Directed evolution	
*Wild type ADH*	*C. glutamicum* R AdhA	Class I	0.29 ± N.I.	0.20 ± 0.01	97 ± 9.8	WT	Wu et al. (2016)
	*L. sphaericus* C3-41	N.I.	0.0029 ± N.I.	N.I.	N.I.	WT	Müller et al. (2015)
	*L. fusiformis* ZC1	N.I.	0.0038 ± N.I.	N.I.	N.I.	WT	
	*B. coagulans* 36D1	N.I.	0.0058 ± N.I.	N.I.	N.I.	WT	
	*D. hafniense* Y51	N.I.	0.0018 ± N.I.	N.I.	N.I.	WT	

N.I.—No information.

NAD^+^-dependent Mdhs from *B. methanolicus* that has been studied a lot (Vonck et al., 1991; De Vries et al., 1992; Hektor et al., 2002; Krog et al., 2013; Ochsner et al., 2014; Müller et al., 2015; Witthoff et al., 2015). They support cell growth and methanol uptake with high speed in native *B. methanolicus*. However, the catalytic activity of Mdhs from *B. methanolicus in vitro* and *in vivo* are limited because of the unclear mechanism of ACT (Hektor et al., 2002; Witthoff et al., 2015), even though ACT significantly improve the *K*_M_ value of Bmmdh (reduced from 1.8- to 14.0-fold) (Krog et al., 2013; Ochsner et al., 2014). Second, an ACT-independent Mdh from *C. necator* was developed and characterized for the kinetics and substrate specificity on 2016 (Wu et al., 2016). It showed the low affinity to methanol (132 mM for *K*_M_) compared to that of Mdhs from *B. methanolicus* (170–360 mM for *K*_M_) (Krog et al., 2013; Ochsner et al., 2014; Müller et al., 2015; Wu et al., 2016). Another study showed an Mdh from *B. stearothermophilus*, which shares 21–23% amino acid identity with the Mdh from *B. methanolicus* (Whitaker et al., 2017). The affinity of Mdh from *B. stearothermophilus* showed a lower value than that from Bmmdh and Cnmdh (20 mM for *K*_M_), thus, it had superior performance *in vivo* than previously published Mdhs. In particular, Lee et al. found an Mdh from *L. xylanilyticus*, that had higher substrate specificity towards methanol than Bmmdh, Cnmdh and Bsmdh (Lee et al., 2020). In addition, it is also an ACT-independent Mdh with an impressively low affinity (3.23 mM for *K*_M_).

To improve the activity of Mdhs, site-directed (Hektor et al., 2002; Ochsner et al., 2014), site-saturation (Wu et al., 2016) or random mutagenesis (Le et al., 2021) is used for creating Mdh mutants. In 2002, Hektor et al. used site-directed mutagenesis to confirm the role of various amino acid residues in the NAD(H) binding site in Mdh from *B. methanolicus* C1 (Hektor et al., 2002). All mutants are impaired in cofactor NAD(H) binding, though, some mutants (G95A, S97G, and S97T) retained Mdh activity. Finally, only the S97G mutant displayed as “fully activated” in Mdh reaction rates. Another study from Ochsner et al. investigated the effect of site-directed mutations in the predicted active site of Mdh from *B. methanolicus* MGA3 (Ochsner et al., 2014). The V_max_ of Bmmdh S98G increased two-fold compared with that of its wild-type (WT), yet its *K*_M_ value also increased in the absence of ACT. Even upon adding ACT, the catalytic efficiency of Bmmdh S98G was similar to that of WT (a doubling of V_max_ with a slight reduction in *K*_M_). Meanwhile, Bmmdh2 S101G lost the activity on methanol. For Mdh from *C. necator*, the site-saturation mutagenesis on the Mdh2 A169 site was constructed (Wu et al., 2016). In the first round of screening, eight possible positive variants with over 50% activity improvement (based on the Nash reaction) were selected from 2000 screened variants, and, finally, CT1-2 was used as the template for another error-prone PCR library in the second round of screening. Afterward, CT4-1, the recombinant of three mutations (A169V, A31V and A26V), which showed a low *K*_M_ (21.6 mM) and an unchanged *k*_cat_ (0.2 s^−1^) compared with WT Mdh2, was created by various rounds of high throughput screening (HTS). For studying the activity of Mdh from *L. xylanilyticus*, eight residues within 4.5 Å of the center of the docked substrate were selected to contribute toward site-directed mutagenesis (Lee et al., 2020). Finally, the mutations S101V (*K*_M_ = 10.35), T141S (*K*_M_ = 51.24) and A164F (*K*_M_ = 36.83) improved the enzyme’s specific activity towards methanol compared to that of the Lxmdh WT. Furthermore, a random mutant library of *L. xylanilyticus* Mdh was constructed and high throughput screened by an formaldehyde detectible biosensor (Le et al., 2021). As a result, several mutants were characterized by high catalytic efficiency and low *K*_M_ compared with Lxmdh WT and its published mutants. Thus, mutant Lxmdh E396V, which has the highest catalytic efficiency (79-fold that of WT catalytic efficiency) and an impressive *K*_M_ value (0.01 mM), was found. Moreover, the *K*_M_ value of another Lxmdh mutant, K318N, was also impressive (0.046 mM). Nevertheless, the recombinant of two mutations (E396V and K318N) had a higher *K*_M_ value compared with each mutant (0.233 mM).

Many alcohol dehydrogenases (ADHs), which can catalyze methanol oxidation, may be treated as Mdhs. Although, the catalytic efficiency of methanol oxidation by ADHs is low, it is another good candidate for synthetic methylotrophy. As an example, the AdhA from *Corynebacterium glutamicum* R has shown a low *K*_M_ value of methanol activity (97 mM) compared with Bmmdh and Cnmdh (Kotrbova-Kozak et al., 2007; Wu et al., 2016). A number of ADH enzymes has been tested for the methanol oxidation activity without kinetic values, such as ADHs from *Lysinibacillus sphaericus*, L*ysinibacillus fusiformis*, *Bacillus coagulans* and *Desulfitobacterium hafniense* (Müller et al., 2015).

Furthermore, critically, improving methanol oxidation rates by kinetically improved Mdh variants would only be enabled in cells where there is sufficiently fast of formaldehyde assimilation (Woolston et al., 2018). This is important for the development of Mdh-directed evolution approaches. This is covered in the synthetic methylotrophy section of this review.

## Application of Mdhs in Synthetic Methylotrophy

C1 feed stocks are inexpensive abiotic resources for microbial bio production. Among all C1, the soluble C1 substrates, such as methanol, may be more suitable feed stocks because of the avoidance of mass transfer limitation (Claassens et al., 2019). Synthetic methylotrophy using the integration of Mdhs for the assimilation of methanol as a carbon source into non methylotrophs such as *E. coli* and *C. glutamicum* has been investigated further in recent studies.

For the design of synthetic methylotrophy, a number of biochemical and practical considerations should be considered. Compared to PQQ-dependent Mdhs and O2-dependent Aods, NAD-dependent Mdhs require only enzyme for its functional assembly in both aerobic and anaerobic conditions. Although, PQQ-dependent Mdhs has very high substrate affinity and activity toward methanol, PQQ biosynthesis requires molecular oxygen (Velterop et al., 1995), which will restrict the applications of PQQ-dependent Mdhs as some of metabolites must be produced only under anaerobic conditions. Unfortunately, there are no PQQ biosynthesis pathway in *E. coli* and *C. glutamicum* as candidate for synthetic methylotrophy. NAD-dependent Mdhs can be utilize a ubiquitous cofactor (NAD) that can be generate reducing equivalents in the form of NADH and used to provide electron for metabolite production under both aerobic and anaerobic conditions and generate reducing equivalents (NADH), which can help promote strain growth. In this point, NAD-dependent MDHs may be the best candidates for synthetic methylotrophy (Zhang et al., 2017).

For instance, introducing NAD^+^-dependent Mdhs is the simplest way to engineer methanol oxidation for all reasons mentioned above. Many researchers are also trying to improve the methanol bioconversion efficiency of synthetic methylotrophy by searching for the NAD^+^-dependent Mdhs with better characteristics from different organisms via directed evolution (Table 3). The Mdhs from *B. methanolicus* (Müller et al., 2015; Witthoff et al., 2015; Dai et al., 2017; Meyer et al., 2018; Tuyishime et al., 2018; Hennig et al., 2020), *B. stearothermophilus* (Whitaker et al., 2017; Bennett et al., 2018; Tuyishime et al., 2018; Bennett et al., 2020; Rohlhill et al., 2020), and *C. necator* (Chen et al., 2018; Tuyishime et al., 2018; Woolston et al., 2018; Chen et al., 2020; Keller et al., 2020) have been used for synthetic methylotrophy in recent studies with *E. coli* as the most popular host (Müller et al., 2015; Whitaker et al., 2017; Bennett et al., 2018; Chen et al., 2018; Meyer et al., 2018; Woolston et al., 2018; Bennett et al., 2020; Chen et al., 2020; Keller et al., 2020; Rohlhill et al., 2020), besides *C. glutamicum* (Witthoff et al., 2015; Tuyishime et al., 2018; Hennig et al., 2020) and *S. cerevisiae* (Dai et al., 2017). *In vitro* system to mimic synthetic methylotrophy using scaffold system by enzyme assembly for enhancement of methanol utilization have been also attempt (Price et al., 2016).

**TABLE 3:** Strategies and advancements in improving methanol bioconversion efficiency of synthetic methylotrophy in recent literature.

Host	Carbon source/substrate	Used Mdh	Refs
*E.coli*	0.4% glucose and 1 M methanol	Mdh from *B. methanolicus* MGA3 and PB1	Müller et al. (2015)
	5 mM sodium gluconate, 20 mM sodium pyruvate, 0.1 g/L yeast extract and 500 mM methanol	Mdh from *B. methanolicus* PB1	Meyer et al. (2018)
	60 mM methanol and 1 g/L yeast extract	Mdh from *B. stearothermophilus*	Whitaker et al. (2017)
	250 mM methanol, 10 g/L glucose		Bennett et al. (2020)
	60 mM methanol and 0.5 g/L yeast extract or 4 g/L glucose		Bennett et al. (2018)
	100 mM methanol and 0.5 g/L yeast extract		Rohlhill et al. (2020)
	6 g/L xylose and 250 mM methanol	Mdh 2 from *C. necator* N-1	Woolston et al. (2018)
	250 mM methanol, 50 mM ribose or xylose, 0.05% casamino acids	Mdh 2 CT4-1 from *C. necator* N-1	Chen et al. (2018)
	400 mM methanol and 20 mM xylose		Chen et al. (2020)
	500 mM methanol and 20 mM pyruvate		Keller et al. (2020)
*C. glutamicum*	120 mM methanol and 55 mM glucose	Mdh and MD3 from *B. methanolicus* MGA3	Witthoff et al. (2015)
	500 mM methanol and 20 mM co-substrates (ribose, xylose or gluconate)	Mdh from *B. methanolicus*	Hennig et al. (2020)
	96.90 mM methanol and 25.32 mM xylose	Mdh from *B. stearothermophilus*	Tuyishime et al. (2018)
		Mdh 3 from *B. methanolicus* MGA3	
		Mdh 2 CT4-1 from *C.* *necator* N-1	
*S. cerevisiae*	10 g/L methanol, 20 g/L glucose, 10 g/L yeast extract and 20 g/L peptone	Mdh from *B. methanolicus* MGA3	Dai et al. (2017)

Although, NAD-dependent MDHs are their favored MDHs for synthetic methylotrophy according to the recent study, the PQQ MDH XoxF has revealed novel activities, such as the oxidation of formaldehyde *in vivo* (Pol et al., 2014; Good et al., 2019). This shows that these enzymes also can generate novel activities for synthetic methylotrophy, even if PQQ must be added; and further, these enzymes may yet reveal undiscovered activities that cannot be generated by NAD-dependent MDHs that would be of great interest to the field.

This consideration could be extended to other steps for engineering synthetic methylotrophy. As mentioned, the speed of formaldehyde assimilation has a big effect on improving methanol oxidation rates. For example, Whitaker et al. combined NAD^+^-dependent Mdh from *B. stearothermophilus* and RuMP pathway enzymes from *B. methanolicus* to engineer *E. coli*, which can grow with methanol as the carbon source. Through their engineered *E. coli* strain (BW25113 ∆*frmA* expressing *B. stearothermophilus* Mdh and *B. methanolicus* RuMP), the amount of biomass derived from methanol was determined to be 0.289 ± 0.028 gCDW/gMeOH in media, including 60 mM methanol and 1 g/L yeast extract. A similar increase of biomass in the presence of yeast extract and methanol at a larger scale was confirmed by bioreactor experiments (0.344 ± 0.012 gCDW/gMeOH) (Whitaker et al., 2017).

## System Biology Based Pathway Optimization

System-wide consideration of engineering strategies is necessary. To address the complexity and identify the best combination of genes for a given host, several computational tools have been developed for the *in silico* design of metabolic pathways (Medema et al., 2012; Vieira et al., 2014; Carbonell et al., 2016). They help identify the best combinations of genes and pathways and optimize the host metabolism, such as transport, cofactors, C1 acceptor regeneration, and chemical toxicity. Müller et al. used the OptFlux software for *in silico* modeling approaches to test the preferred choice of enzymes and pathways by modifying a stoichiometric genome-scale *E. coli* model. A model containing 1,271 gene products and reactions with 1,676 metabolites was established and modified to find a solution for efficient methanol metabolism as a carbon source with a maximal µ of 0.88 h^−1^ (Müller et al., 2015). Later, in 2018, Meyer et al. performed reaction knockout (KO) analyses using FlexFlux based on the *E. coli* models iAF1260 and iML1515 containing additional reactions for NAD^+^-dependent Mdh, HPS and PHI (Meyer et al., 2018). As another example, Keller et al. used cobra python for flux balance analysis (FBA) of the core metabolism of an *E. coli* model from BiGG (Keller et al., 2020).

In methylotrophs, the absence of methanol (or formaldehyde) controls the expression of genes involved, so microorganisms can adapt to the changing of carbon sources (Selvamani et al., 2017). For this reason, regulating the gene expression of methanol and the formaldehyde response is also important. Another important factor is the efficient regeneration of formaldehyde acceptors for methanol assimilation (Woolston et al., 2018). In this regard, it is worth mimicking native methylotrophs (Wang et al., 2019). Five enzymes of the nonoxidative pentose phosphate pathway (PPP) from *B. methanolicus* were introduced into *E. coli* (Bennett et al., 2018). The whole PPP is usually kept for formaldehyde acceptor regeneration, however, it prevents methanol consumption in the absence of a cosubstrate (such as glucose). Therefore, synthetic methanol-dependent strains are engineered for methanol as a co-consumption regime. This leads to the cell growth being bound to methanol assimilation to improve methanol utilization via adaptive laboratory evolution (ALE) (Chen et al., 2020; Wang et al., 2020).

## Conclusion

In this review, the enzymatic properties of various reported Mdhs and their applications in synthetic methylotrophy were discussed. Protein engineering and molecular modifications using site-directed mutagenesis, random mutagenesis, HTS, and direct evolution can potentially advance further studies in this field by improving the properties (i.e., activity, thermos ability, and substrate-binding affinity) of existing Mdh enzymes and discovering new Mdh enzymes. The proposal for engineering Mdh-based synthetic methylotrophy is providing value-added products from methanol. Until now, several useful metabolites of methanol have been produced, proving the potential of methanol-based bio-manufacturing. Therefore, we may take advantage of Mdhs for the utilization of methanol as feedstock for high value chemicals, which is a methanol-based bio economy.
